# The built environment impacts on route choice from home to school for rural students: A stated preference experiment

**DOI:** 10.3389/fpubh.2022.1087467

**Published:** 2022-12-07

**Authors:** Li Han, Yan Wang, Yibin Ao, Xuan Ding, Mingyang Li, Tong Wang

**Affiliations:** ^1^College of Environment and Civil Engineering, Chengdu University of Technology, Chengdu, China; ^2^Department of Engineering Management, Sichuan College of Architectural Technology, Chengdu, China; ^3^College of Management Science, Chengdu University of Technology, Chengdu, China; ^4^Faculty of Architecture and the Built Environment, Delft University of Technology, Delft, Netherlands

**Keywords:** rural school travel built environment, stated preference survey, school travel, multinomial logit model, experiment

## Abstract

**Introduction:**

Rural roads and built environment in China have been developed enormously, but it is not clear whether these roads fulfill the needs of school children as they need to travel long to school every day.

**Objective:**

It is crucial to understand the influencing factors of their travel mode choices to better design future country roads and built environment, aiming to promote physical activities of school children in a safe built environment.

**Method:**

This study thus attempts to explore the impacts of rural built environment attributes on children's school travel mode preferences. Eight rural built environment attributes are considered: distance from home to school; the number of intersections passed on the way to school; whether there are sidewalks/bicycle lanes; the traffic speed of school access routes; whether there are separation facilities between motor vehicles and non-motor vehicles; whether there are traffic lights and zebra crossings; availability of greenery such as lawns, flower ponds and street trees and whether there are shops on the way to school and at the school gate. Six hundred and thirty eight valid questionnaires were obtained through face-to-face interviews with school-age children in villages. A multinomial logit model was estimated to unravel the preferences and choices of rural school-age children in different models of school travel using the stated choice data.

**Results:**

All the eight attributes have significant impacts on rural children's school travel choices on foot, bicycle, electric bicycle or motorbike. And four rural road design attributes have significant effects on rural children's school travel by private cars. A travel path with pavements or bike lanes, few intersections, low traffic speeds, greenery and shops can facilitate children's school travels on foot or by bike. The conclusions can provide reference for the further upgrading planning, designing and construction of rural roads, as well as enriching the theory and practice of child-friendly villages construction.

## Introduction

**Figure 1:**
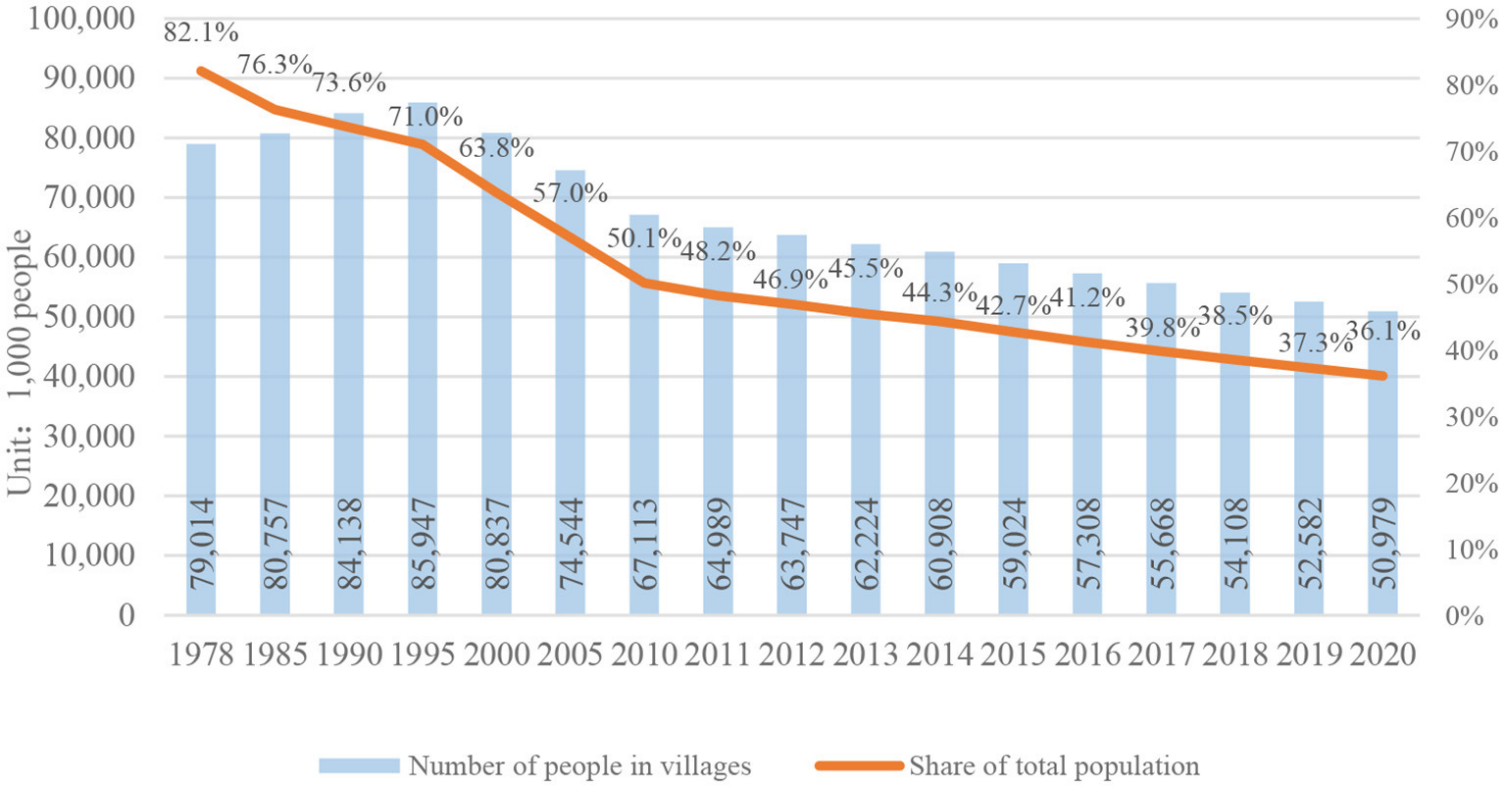
Number and proportion of the country's population living in rural areas.

At present, China has entered the stage of rapid development of new countryside and urbanization. Since 1995, the number of rural population in China has shown a gradual downward trend. According to the data of the seventh population census in China Rural Statistical Yearbook 2021, the number of rural population in 2020 will only account for 36.1% of the total population in China (1) (See Figure 1).

While the rapid urbanization of the countryside has contributed to its rapid development, it has also given rise to a series of problems, such as the destruction of the ecological environment due to the construction of roads, the overcrowding of the urban population, the inadequate supply of housing for low-income people in the cities, traffic safety and traffic congestion, etc. The original housing structure and transport structure of rural residents have also changed accordingly with the urbanization process (2).

**Figure 2:**
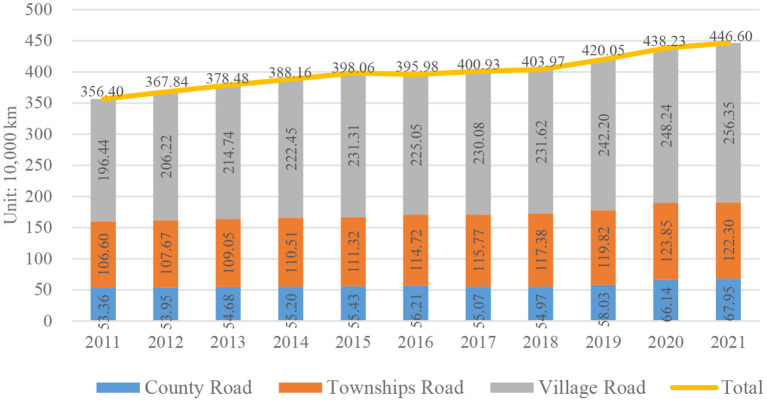
Mileage of national rural roads.

With the rapid development of China's infrastructure and economic growth prompting the rapid development of rural road construction, The mileage of rural roads in China has increased significantly (3) (See Figure 2), rural roads cover both urban and rural areas, and household car ownership in rural areas of China has also changed dramatically in the past two decades or so (4). However, there are still many rural roads with weak traffic infrastructure (such as lack of greening, improper design of traffic lights and zebra crossings). At the same time, rural residents' weak awareness of traffic safety has also brought many safety problems.

**Figure 3:**
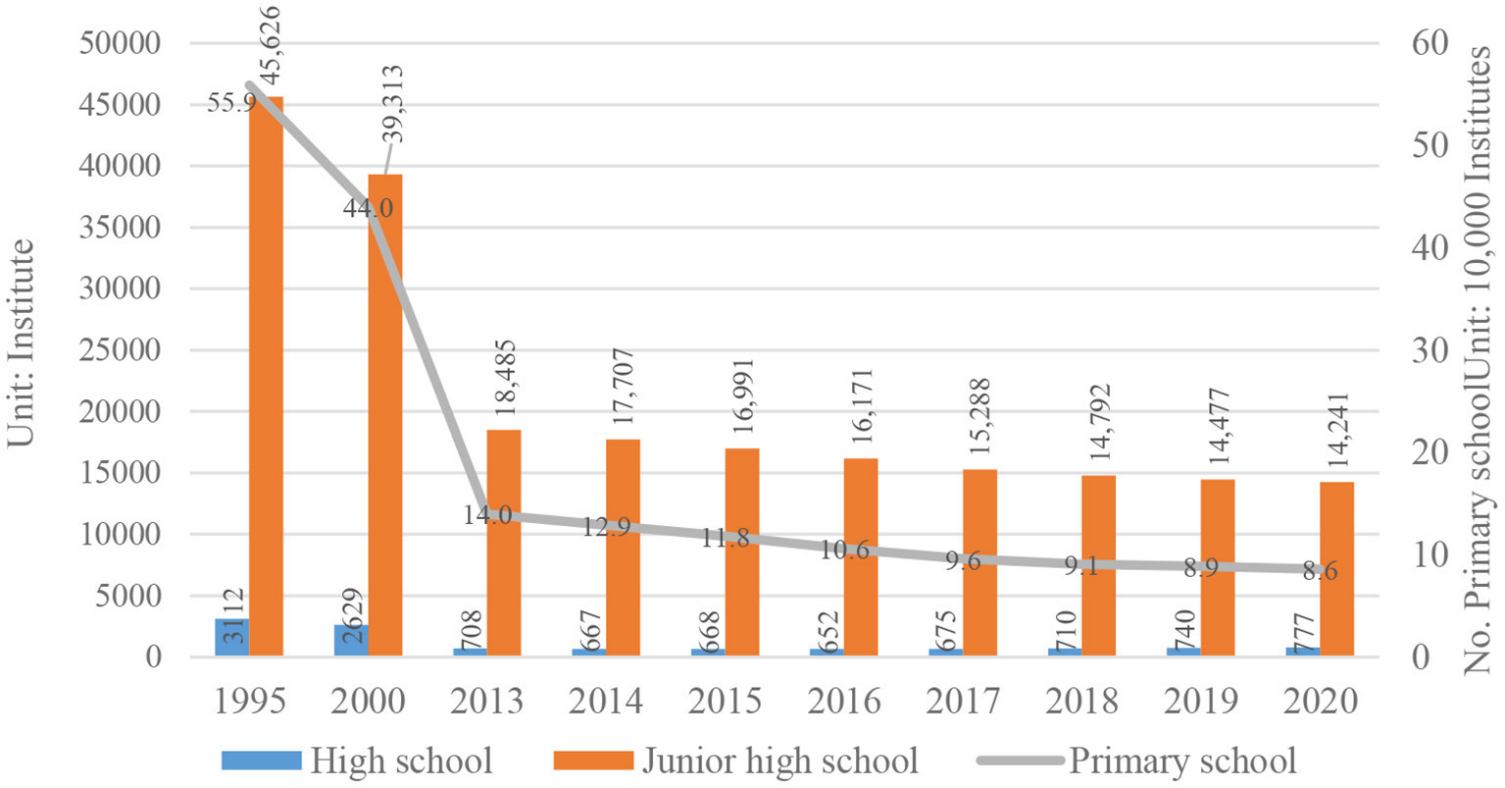
Number of rural schools in China.

**Figure 4:**
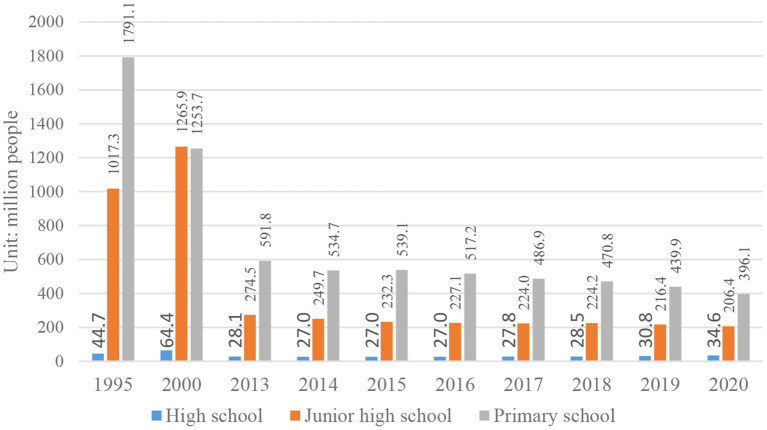
Enrollment of rural schools in China.

Although the rural population in China is on the decline, according to the literature and previous rural research experience, the Chinese school-age children group still has a certain scale, which has generated huge travel demand. On the other hand, the number of rural schools has shown a trend of reduction due to concentration. At the beginning of the 21st century, the Ministry of Education of China launched the School Map Reorganization (SMR) plan, which involved closing small rural schools, opening large centralized schools in townships and counties, and shifting from “running schools in villages and villages” to integrating the resources from nearby schools. According to statistics, in the 20 years from 2000 to 2020, more than 350,000 primary schools have been canceled or merged. By the end of 2020, there are only 777 high schools, 14,241 junior high schools and 86,000 primary schools in rural China (See Figure 3), and the number of students enrolled has also decreased year by year (1) (See Figure 4).

The sharp drop in the number of schools due to the SMR plan has caused many children to lose the chance to study in their local schools (5). Some rural students and a small number of urban students can only choose boarding schools or take private cars to/from school to overcome the increasing home-to-school distance. All of these led to changes in the way of school travel (6).

The World Health Organization has made a survey and clearly pointed out that the number one cause of children's death in the world every year is traffic accidents, which accounts for a higher proportion in China. Globally, about 5 million people die each year from road traffic accidents. More than 70% of them occur in developing countries. And 80% of these accidents are related to children. This has become a serious social problem. In recent years, school age children have frequent traffic accidents. According to the statistics of China's transportation department, more than 18,500 children die in traffic accidents every year in China. This mortality rate is 2.5 times that of European countries and 2.6 times that of the United States (7).

It is crucial to understand the preferences of children in rural areas and create a well and safe built environment for them to promote public health and active travels like walking and cycling. Most previous studies have explored the relationship between general built environment attributes and residents' preferences on different spatial scales, as reviewed by Saelens and Handy (8), Ikeda and Stewart (9), and Chen et al. (10), but failed to capture the micro-scale environmental factors like the detailed values of crossings on a road, the numbers of shops, etc. This research therefore aims to quantitatively study the relationship between children's preference and the micro-scale attributes of built environment travel paths for better design of the school path. In this study, declarative choice experiments were used to explore children's preferences, and discrete choice models were used to estimate the quantitative relationship between micro built environment attributes (i.e., in this study eight travel path attributes) and children's overall preferences for travel modes.

Context-wise, at present, Chinese scholars have done much research on medium and large urban areas, but little research on rural children's school travel, especially in southwest China. Therefore, this study takes into account the current situation of rural development and the living habits of rural residents, and examines the attributes that influence children's school travel behavior and their influential relationships, and proposes practical strategies for planning and building child-friendly school travel roads in rural areas.

The rest of the paper is organized as follows: In Section Literature review, the selection of experimental design and data collection for the study are discussed. Section Stated choice design and data collection provides a discussion of the results of the model. Finally, section Results and discussion gives conclusions and policy recommendations.

## Literature review

School travel is the main daily activity of children, and is also an important part of the daily life of many families. It mainly refers to the behavior of students commuting between school and home. The current modes of school commuting include walking, cycling, bus, car, public transportation, etc. Many countries refer to walking and cycling as “an active way of traveling,” which is feasible in children's daily life and can increase children's physical health.

The factors affecting children's choice of school travel mode mainly include personal factors, family factors, travel characteristics factors, built environment factors and other factors.

Scholars studied on travel behavior earlier and made many achievements. At the same time, their focus gradually shifted from adults to children since 1980. The research on children's school travel behavior through school started late in China, and the research results are also relatively limited. In the existing literature, research on personal factors mainly includes age (11–14), gender (13, 15–18), race (19–21), etc. Family factors mainly include family income (22–25), family car ownership rate (26–29), family driver's license ownership rate, parents' convenience of transportation (12, 30, 31), parents' attitude toward students' travel (16, 32–34), number of brothers and sisters (32, 35), parents' education level (20, 29) and other family factors. The impact of travel characteristic factors on students' choice of commuting mode is mainly reflected in travel distance (36–42) and travel duration (28, 40). Compared with the first three types of factors, the built environment factors started late in the children's school travel and included a wide range of contents, so the impact on the choice of school age children's way of integration is also more complex. Traffic infrastructure conditions, the number of intersections, community resources, etc. will have an impact on children's school travel. For example, high pavement integrity of pedestrian roads (43), high bicycle lane coverage (44), and high shade tree density (45) all promote children's active school travel, while the increase in the number of street areas and intersections is not conducive to children's independent walking to school (45). Ikeda's research has shown that children's active school travel is positively related to their neighborhood safety perception. Obviously, compared with measurable objective factors, children's subjective perception of the environment is equally related to their learning behavior (34). In summary, the common factors impacting children school travel mode choices in the existing research include residential density (46–48), residential location (49, 50), pedestrian facilities (28, 51), travel safety (29, 52), etc.

Furthermore, Müller et al. (53) found that the closure of some schools (similar to China's policy of “removing points and merging schools”) had a negative impact on the way students go to school. Khan et al. (54) survey of factors influencing students' choice of transportation to school revealed that students were more sensitive to factors such as the cost, time, and comfort in the school bus when choosing a school bus to school. Weather is also a major obstacle to children's active travel (55, 56). In addition to this, Grize et al. (57) found that cultural factors influence children's travel mode choice, and Dalton et al. (58) found a correlation between the frequency of active school travel and season in a study of rural children.

By retrieving relevant research literature on influencing factors of school age children's school travel, it is found that in recent years, scholars have discussed the travel characteristics of students in various countries and regions and the influencing factors of their travel modes, and gradually focused on the impact of built environment on children's school travel.

The combination of mature theoretical research and successful practical experience has an important guiding role in studying the influencing factors of Chinese rural children's school travel modes and improving the built environment for them. However, the influencing factors and related conclusions obtained by scholars from other contexts are not necessarily consistent with China's national conditions and cannot be directly copied.

Chinese scholars' research on children's travel modes and influencing factors is mostly concentrated in Hong Kong (49), Beijing (59), Shanghai (60) and other large cities, and some research on small and medium-sized cities (61), with little research for rural areas.

Although rural areas have been developing rapidly in recent years, there is still a large gap between urban and rural areas in terms of built environment, school layout and road scale (62). The research conclusions on the impact of urban built environment on children's school travel behavior cannot be fully applicable to the vast rural areas. Currently, Chinese scholars have conducted relatively few studies on children's travel to school in rural areas and the influencing factors exist, and these studies are still in the exploratory and initial stages, and rarely for the southwest of China. In-depth research should be conducted on the factors influencing children's school travel, taking into account the current state of rural development and the living habits of rural residents in southwest of China.

## Stated choice design and data collection

### Attribute selection

More specifically, for the travel path built environment attributes' impacts on travel mode selection, some scholars have found that distance has a great impact on transportation mode choice (17, 63–65), and the distance between home and school has a strong negative impact on the choice of walking to school (66). Otherwise, high traffic speeds (especially more than 30 km/h) and volume increase the risk of serious or fatal injuries to children and pedestrians, and may prevent parents from encouraging their children to walk to school (55, 67).The increase in the number of street areas and intersections also negatively affect children's independent walking to school (45).

For Chinese children in rural areas, parents often are concerned that their children will be involved in traffic accidents when walking in areas that lack facilities such as sidewalks. Scholars believe that the implementation of effective pedestrian interventions can reduce the traffic risks that hinder children from walking to school (68). For example, children have a higher proportion of walking to school in the built environment with high pavement integrity (43, 69).

Traffic lights and bicycle infrastructure are one of the main attributes that encourage cycling (70). Bicycle-specific facilities (SBFs), such as safety islands, raised curb pavement and other infrastructure separating motor vehicles and bicycles, can improve the safety of cycling, and such facilities play a good role in promoting cycling (68). Lin's observational study of adolescents found that high shade tree density and high sidewalk coverage encouraged children to walk to school independently (45), and Bosch's study found that the average density of convenience stores along the way was positively correlated with high positive traveling rate (71).

Therefore, on the basis of existing literature research and considering the actual situation of rural construction in Chengdu (in Southwest of China), this study selected eight built environment attributes, namely distance, number of intersections along the school path, sidewalk/bicycle lane, traffic speed, separation facilities between motor vehicles and non-motor vehicles, traffic lights and zebra crossings, green plants along the school path, and shops.

To design these eight attributes value levels, existing studies and the rural area conditions are combined to determine the tailor-made attribute levels for this research.

Several studies by scholars in other countries have shown that distance tends to be the primary concern of most parents. For most students, 1 mile is the maximum walking distance (72), and 2 miles is the maximum cycling distance (73). In addition, Kontou pointed out that walking was the most common way of school travel in urban and rural areas when the distance to school was < 0.5 mile, and the bicycle riding rate peaked when the home-school distance was between 0.5 and 1 mile (38).

Based on the previous research experience and data collection in nearly 100 rural areas in Sichuan Province (where the case area is located), the distance attribute in this study is divided into four levels: < 0.5, 0.5 to 1 km, 1 to 2.5 km, and more than 2.5 km. At the same time, we set the attribute level of the number of intersections passing through as: >5, 3–5, 1–2, and 0.

Considering the different development conditions in rural areas and the limited awareness of rural children on traffic facilities; it is impossible to accurately quantify the traffic speed of rural school paths, lawn, flower beds, street trees and other green plants and shops on the way to school. Therefore, the level of five attributes of green plants such as sidewalk/bicycle lane, separation facilities between motor vehicles and non-motor vehicles, traffic lights and zebra crossings, green plants on the way to school, and shop is set as Yes or No, so as to facilitate the children interviewed to judge and answer.

The traffic speed of the school path is set to two levels: high speed (≥30 km/h) and low speed (< 30 km/h). The attribute design level values are shown in Table 1.

**Table 1:** Attribute levels.

**Attributes**	**Levels**			
**Distance**	** < 0.5 km**	**0.5–1 km**	**1–2.5 km**	**>2.5 km**
Number of intersections passed	>5	3–5	1–2	0
Sidewalks/bike lanes	Yes		No	
Traffic speed of school path	High speed (≥30 km/h)		Low speed (< 30 km/h)	
Machine non isolation facilities	Yes		No	
Traffic lights and zebra crossings	Yes		No	
Green plants	Yes		No	
Shops	Yes		No	

### Experimental design

There are many attributes that affect children's choice of school travel mode. Compared with judging directly a specific road segment, the statement choice experiment can control the covariance of the attribute level. Therefore, when other conditions are the same, the results reflect a more basic measure of children's preferences.

The application of stated choice experiments involves the creation of a design that combines the attribute levels in a particular manner. In this study, the two attributes with four levels and six attributes with two levels would result in 4^2^ × 2^6^ different profiles in a full factorial experimental design that involves all possible combinations of attribute levels. In order to make the questionnaire concise and reduce the dependence between different variables, the experimental design method was used for scenario combination. Orthogonal design and uniform design are two commonly used experimental design methods. However, orthogonal design was applied in the questionnaire because it could be used for experiments with a small number of levels ( ≤ 5).

In this study, Statistical Analysis System (SAS) statistical software was used to orthogonal combine eight attributes and corresponding levels in the design, and an orthogonal fractional factorial containing 32 attribute profiles was selected. Choice sets were created by randomly combining these 32 attribute profiles, thereby creating choice sets of two unlabeled alternatives. Each option added the option of “None” to allow the possibility that both options are lower than a certain selection threshold. The 32 choice sets were divided into eight blocks to reduce the burden on the respondents. In the end, each interviewee needed to answer the questions in four scenarios in a questionnaire. In each scenario, there are four different ways of school travel. The modes of transportation are: walking, bicycle, electric bicycle/motorcycle, and private car. The interviewees were asked to choose their favorite route scenario under each mode of transportation. If the interviewees chose “none,” it means they did not like any of them. Table 2 shows an example of a selection set.

**Table 2:** An example of a stated choice set for children.

	**Street profile A**	**Street profile B**	**None**
Distance	0.5–1 km	0.5–1 km	
Number of intersections passed	0	1–2	
Sidewalks/bike lanes	No	No	
Traffic speed of school path	Low speed	High speed	
Machine non isolation facilities	Yes	Yes	
Traffic lights and zebra crossings	Yes	Yes	
Green plants	Yes	Yes	
Shops	No	No	
If you walk to school, you choose	[ ]	[ ]	[ ]
If you go to school by bike, you choose	[ ]	[ ]	[ ]
If you go to school by electric bicycle/motorcycle, you can choose	[ ]	[ ]	[ ]
If you go to school by private car, you choose	[ ]	[ ]	[ ]

### Data collection

#### Questionnaire design and survey administration

The questionnaire consists of three parts. The first part briefly introduces the research project and research purpose. The second part includes relevant variables of social population and rural children's school travel. The socio-demographic information involves children's gender, age, grade and whether they are the only children in the family. In order to supplement the information about children's families, the parents of the children interviewed were investigated by socio-demographic statistics. The variables include: gender, age, personal and total family income, education level, whether or not having a driving license, the number of private cars, motorcycles, electric bicycles and bicycles held by families. The third part includes four Stated Preference (SP) multiple-choice questions. The SP survey part of each questionnaire contains four combination scenarios, with a total of 16 designated choice questions. Under the premise of four modes of transportation: walking, cycling, electric bicycle/motorcycle, and private car, they can choose their preferred path to school.

**Figure 5:**
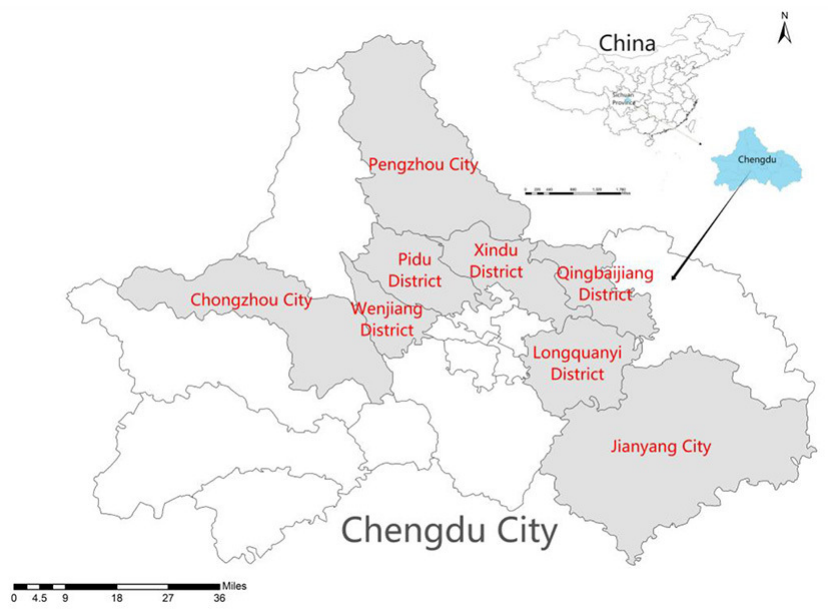
Distribution map of investigation areas.

Because children have not been exposed to such surveys, they may have misunderstanding and choose wrong options. Therefore, before asking the children to complete these 16 tasks, the investigators showed the children interviewed photos or videos of attributes involved, and explained all attributes and their levels. Then, the investigators made an example of selection to help the children to understand. The research team has organized a face-to-face questionnaire survey with rural children in July 2021. The preliminary selection of sample areas and villages was carried out by collecting information online and looking up maps. According to the distance from the center of Chengdu, the suburban areas were selected: Wenjiang District, Xindu District, Longquanyi District, Pidu District; Chongzhou City, Qingbaijiang District, Jianyang City, and Pengzhou City. The sample villages are selected from the eight regions. The regional distribution is shown in Figure 5.

Investigators learn about the situation of each village by communicating with residents, querying information online or searching maps, and select villages that are convenient, well-developed, and have schools in the village or nearby villages. The researchers also select several nearby villages to prevent insufficient sample size. Before the formal investigation, we conducted a pre-investigation, and selected 33 sample villages randomly according to the vigilance and cooperation of the villagers. The respondents all have agreed their participation in the research and all the data has been anonymized. For non-adults, their guardians have approved their participation as well.

#### Sample characteristics

Six hundred and thirty eight valid questionnaires were finally collected through household survey in 33 villages. In this study, the dependent variable travel mode is defined as walking, bicycle, bus, private car, electric bicycle and motorcycle. Among them, the proportion of choosing electric bicycle is the largest, 58.60%, followed by private cars, pedestrians and buses, 14.10, 13.20, and 9.20%, respectively. The proportion of students who choose bicycles and motorcycles for school travel is relatively less, 3.40 and 1.40%, respectively.

The detailed information of the selected socio-demographic variables' used in this research are shown in Table 3. According to the survey data, most of the surveyed children are boys, with 339 in total, accounting for 53.1% of the total surveyed children. 67.4% of the surveyed children are at the grade level of primary school. Most of the rural areas have built rural or township primary schools. It is easy for rural children to go to primary school, while rural school-age children may go to school in different places because of school choice when they go to junior high school. 52.2% of the surveyed children's parents are over 50 years old, and more than half of the parents are grandparents of the surveyed children. 51.3% of the surveyed parents have education level in primary schools and below. It can be seen that young and middle-aged workers are more willing to go out to work, while grandparents stay in the village to take care of children's life and study. The annual personal income of 48.2% of the interviewed parents was < 10,000 RMB, and the percentage of children with total family income between 10 and 50,000 RMB was 46.0%. With the continuous promotion of the rural revitalization process and the development of the rural economy, 47.8% of the families owned a private car. The proportion of households with one electric bike was the highest, followed by private cars, bicycles and motorcycles. Compared with motorcycles, rural residents are more willing to choose light, flexible and affordable electric bikes for daily travel.

**Table 3:** Socio-demographic variables.

**Personal attributes**		**Frequency**	**Personal attributes**		**Frequency**
Gender	Male	53.1%	Only child	Yes	62.9%
	Female	46.9%		No	37.1%
Age	6–12 years old	69.0%	Grade	1–6	67.4%
	13–15 years old	22.0%		7–9	22.7%
	15–18 years old	8.9%		10–12	9.9%
**Parental attributes**		**Frequency**	**Parental attributes**		**Frequency**
Gender	Male	36.1%	Relationship with the children	Father	14.6%
	Female	63.9%		Mother	29.0%
Age	Under 30 years old	5.0%		Grandfather	24.3%
	31–40 years old	31.5%		Grandmother	31.8%
	41–50 years old	11.5%		Other	0.3%
	51–60 years old	35.5%	Annual personal income	< ¥10,000	48.2%
	60 years old or older	16.7%		¥10,000–50,000	39.7%
Education level	Primary school and below	51.3%		¥50,000–100,000	11.1%
	Junior high school	24.3%		¥100,000–150,000	1.6%
	High school or junior college	20.1%	Driver's License	Yes	31.0%
	College degree	2.4%		No	69.0%
	Bachelor's degree	1.9%	Can ride a motorcycle	Yes	89.7%
	Master's degree and above	0.2%	/Electric bicycle	No	10.3%
**Parental attributes**		**Frequency**	**Parental attributes**		**Frequency**
Family members	3 and under	8.8%	Total household income	¥10,000–50,000	30.1%
	4	19.0%		¥50,000–100,000	46.0%
	5	41.1%		¥100,000–150,000	18.4%
	6 and above	31.1%		¥150,000–200,000	5.2%
				More than ¥200,000	0.3%
Number of cars	0	52.2%	Number of motorcycles	0	81.2%
	1	45.9%		1	18.5%
	2	1.7%		2	0.3%
	3 or more	0.2%		3 or more	0
Number of electric bicycles	0	6.0%	Number of bicycles	0	74.7%
	1	83.7%		1	24.8%
	2	8.9%		2	0.3%
	3 or more	1.4%		3 or more	0.4%

### Data coding

After the questionnaire work was completed, the unfilled questionnaires were first screened and valid survey data were entered to test their reliability and validity. In addition, the study also coded the socio-demographic variables and micro-attributes of the built environment for school travel as required by the SP questionnaire data analysis. For example, male gender is coded as 1, female gender is coded as−1. For micro-graphic built environment attributes, three indicator variables were constructed for each attribute with four levels. Each indicator variable corresponds to a category coded as 1, the remaining categories are coded as−1 for all three indicator variables. For each attribute with two levels, two indicator variables are constructed, and the category corresponding to the “yes” indicator variable is coded as 1, and the category corresponding to the “no” indicator variable is coded as -1.

## Results and discussion

Modeling analysis was conducted based on the SP survey to construct a functional relationship between transportation mode and influence attributes. In this study, multinomial logit model (MNL) was used (74) to analyze the preference of the interviewed children for school travel environment under different transportation modes, and all four models passed the chi-square test, and the significance was < 0.05, meaning that the models fit well. And the test results and model fitting parameters are shown in Tables 4, 5, respectively.

**Table 4:** Likelihood ratio test for independent variables.

**Model**	**Chi-square test**	
	**Chi-square**	**Significance**
The model of walking conditions	1523.014	0.000
The model of bicycle conditions	1220.765	0.000
The model of electric bicycle/motorcycle conditions	962.850	0.000
The model of private car conditions	1998.975	0.000

**Table 5:** MNL model parameter estimation.

**Attributes**	**Levels**	**Walk**		**Bicycle**		**Electric bicycle/motorcycle**		**Private car**	
		**Coefficient**	**Prob**.	**Coefficient**	**Prob**.	**Coefficient**	**Prob**.	**Coefficient**	**Prob**.
			**|z|**>Z*		**|z|**>Z*		**|z|**>Z*		**|z|**>Z*
Constant 1	1	−0.23602***	0.0000	0.55352***	0.0000	1.15344***	0.0000	0.19850***	0.0027
Constant 2	2	−0.33153***	0.0000	0.54682***	0.0000	1.05480***	0.0000	0.24947***	0.0002
Distance	< 0.5 km	1.85974***	0.0000	0.73180***	0.0000	−1.42555***	0.0000	−2.68426***	0.0000
	0.5–1 km	0.73208***	0.0000	1.24804***	0.0000	0.24302***	0.0000	−0.70456***	0.0000
	1–2.5 km	−1.17038***	0.0000	−0.01883	0.7530	0.76149***	0.0000	1.27666***	0.0000
	>2.5 km								
Number of intersections passed	>5	−0.20194***	0.0045	−0.30775***	0.0000	−0.26279***	0.0000	−0.69916***	0.0000
	3–5	−0.24675***	0.0002	−0.09207	0.1290	−0.11990**	0.0267	0.02579	0.6998
	1–2	0.03199	0.6113	0.11466**	0.0421	0.00822	0.8837	0.13555*	0.0650
	0								
Sidewalks/bike lanes	Yes	0.15930***	0.0000	0.24438***	0.0000	0.14094***	0.0000	−0.01961	0.6201
	No								
Traffic speed of school path	High speed (≥30 km/h)	−0.10151***	0.0055	−0.37477***	0.0000	−0.20032***	0.0000	0.02775	0.4869
	Low speed (< 30 km/h)								
Machine non isolation facilities	Yes	0.11928***	0.0010	0.22611***	0.0000	0.21501***	0.0000	0.00748	0.8481
	No								
Traffic lights and zebra crossings	Yes	0.09818**	0.0114	0.39925***	0.0000	0.39301***	0.0000	0.27501***	0.0000
	No								
Green plants	Yes	0.19735***	0.0000	0.32175***	0.0000	0.28826***	0.0000	0.23838***	0.0000
	No								
Shops	Yes	0.10227***	0.0077	0.22700***	0.0000	0.16474***	0.0000	0.06695	0.1027
	No								

^***^, **, * indicate significance at 1, 5, and 10% levels, respectively.

### Analysis of rural children's preference of walking in school travel environment

On the premise that walking is selected as the school travel mode, the influence of distance attribute on the first and second levels is 1% significant at the general level, with positive values. This indicates that when children choose to walk to school, they are willing to walk within a distance of < 500 m and a distance between 500 m and 1 km, and the first rank coefficient is greater, indicating that the interviewed children prefer to walk to school within a distance of < 500 m. The influence of distance on the third level is significant and negative at the normal level, which indicates that children do not like walking for the distance between 1 and 2.5 km.

The influence of the number of intersections on the first and second levels is 1% significant at the normal level, with negative values, indicating that children who walk to/from school do not like the sections with the number of intersections between 3 and 5 or more. This is consistent with existing findings that neighborhood built environment characteristics (i.e., major street intersections, retail density, and neighborhood density) are strongly associated with the odds of walking (75–78).

The partial value utility of sidewalks suggests that children who walk prefer the presence of sidewalks, which is consistent with existing research (43). Lack of sidewalks or intermittent sidewalks are considered a barrier because they make walking to school more dangerous, and discontinuous sidewalks force children to cross the street repetitively, increasing the number of children crossing intersections and posing a potential hazard (30). The installation/widening of crosswalks and sidewalk improvements would result in a significant increase in the number of children walking or bicycling to school (71). However, in the actual rural school travel built environment, most rural areas only have sidewalks near school sections, and in some rural areas even all sections are country roads, which indicates the need for sidewalks in future planning.

The effect of the first level of the vehicle speed attribute has a negative significance at the 1% level at conventional levels, indicating that children walking to/from school do not prefer environments with vehicle speeds >30 km/h. This may be due to the fact that a school travel built environment with high vehicle speeds can compromise children's walking safety. Carlson et al. (79) combined the effect of major roads on the odds of walking to school and found that children and parents may seek a route with lower traffic speeds to walk to school. As shown in Table 5, when children walk to school, they prefer facilities with road teeth, fences, green strips, and delineations that separate them from motor vehicle lanes. For children who walk, the effect of having traffic lights and crosswalks was significant at the 5% level, indicating that children who walk to/from school prefer crossing facilities. Greening facilities on the path to and from school indicate that children on foot prefer greenery such as lawns, flower ponds, and street trees on the path. In addition, the study finds that children who walk to/from school prefer to have shops on the way to school or in front of the school, probably because children are younger and like to stay and play at the shops on their way to/from school or buy small items such as snacks, toys, and stationery.

To summarize, for walking to school, the attribute that children care most about is distance, they are most likely to walk to school within 500 m. As the distance increases, their chances of choosing to walk to school decrease. The installation of sidewalk and isolation facilities between motor vehicles and non-motor vehicles will bring a sense of security. The green plants such as lawn and street trees along the school path will provide shade, isolation protection, and increase the sense of visual beauty for children who walk to school, thus promoting children to choose to walk to school, while the high traffic speed will hinder children from choosing to walk.

### Analysis of rural children's preference of cycling in school travel environment

The effects of the distance attribute at the first and second levels were positive 1% significant at conventional levels, given the choice of bicycle to/from school, indicating that when children choose bicycle to pass school, they prefer to ride a bicycle at distances < 500 m and at distances between 500 m and 1 km. Interestingly, the coefficient of the distance attribute is greater at the second level and the effect of the distance attribute at the third level is not significant at the conventional level, indicating that the interviewed children prefer to take the bicycle mode to pass school within the range of 500 m to 1 km. This is similar to the study by Kontou et al. (38) who found that the rate of bicycling peaked at home-school distances of between 0.5 and 1 mile.

The effect of the first of the number of intersections attribute is significant and negative at the conventional level, which indicates that children who cycle to/from school do not prefer road environments with more than five intersections. The effect at the third level is significant at the conventional level of 5%, indicating that children prefer to take the bicycle in a road environment with 1–2 intersections on the way.

Part of the value and utility of bicycle lanes show that children who travel to school prefer the road environment with bicycle lanes. The effect of the first level of the speed attribute had a negative significance of 1% at the conventional level, indicating that children do not prefer environments with speeds >30 km/h when choosing bicycles to get to/from school, which may be due to the fact that a built environment with too high a speed can affect children's safety through school.

The property of the isolation facilities between motor vehicles and non-motor vehicles shows that children prefer separated facilities from motorized lanes when bicycling to school, and that a built environment with separated facilities makes children feel safer.

For children who bicycle to/from school, the effect of having traffic lights and crosswalks was significant at the 1% level with a high significance coefficient, indicating that children who bicycle to/from school prefer cross-street facilities. The greenery on the path to school showed that children who bicycle to school prefer to have greenery such as grass, flower ponds, and street trees on the path to/from school. Children who bike to/from school prefer traveling paths with shops on their way or in front of the school. This is consistent with established research that higher average density of convenience stores is associated with higher odds of active traveling (71).

It is found that the maximum distance that rural children can accept is 1 km, and the distance they are most willing to ride a bike is between 500 m and 1 km. This may be because the distance of 500 m is short and it is not as convenient as walking to park a bike. During the visit, many children also said that they were not allowed to ride bicycles to/from school alone. Even though they had the ability to ride bicycles to/from school independently, parents do not allow children to ride bicycles to school independently for fear of children's safety. This is because that most of the rural roads have many intersections, with no special bicycle lane, and there is a lack of a safe bicycle environment.

### Analysis of rural children's preference of electric/motorcycling in school travel environment

The effect of the distance attribute at the first level was 1% significant at the conventional level and negative when children chose to ride the electric bicycle/motorcycle to/from school, indicating that when children chose to ride the electric bicycle/motorcycle to and from school, they did not prefer to ride the electric bicycle/motorcycle at distances of < 500 m. The effect of the distance attribute at the second and third levels was positively significant at the conventional level, indicating that children preferred to ride the electric bicycle/motorcycle at distances of 500 m or more. The effects of the first and second levels of the number of intersections attribute had a negative significance of 1% at the conventional level, indicating that children who ride to/from school by electric bicycle/motorcycle do not prefer road environments with more than three intersections; and the effect at the third level was not significant at the conventional level.

Some of the value utility of sidewalks/bike lanes suggests that children who go to school on electric bicycle/motorcycles prefer a road environment with bike lanes, possibly because children perceive that electric bicycle or motorcycles can be driven on bike lanes and that bike lanes provide them with safety and convenience.

The effect of the first level of the speed attribute has a negative significance of 1% at the conventional level, indicating that children do not like to choose electric bicycles or motorcycles to pass school in road environments where speeds are generally >30 km/h, and that driving environments with excessive speeds can be dangerous for children. The results show that when they go to school by electric bicycle or motorcycle, they liked to have such isolation facilities as curb, fence, green separation belt and scribing.

The school travel built environment with isolation facilities will enhance children's sense of security. The traffic light and zebra crossing attributes have a 1% level of significance at the first level of impact with a high significance coefficient, which indicates that children who ride to/from school on electric bicycles or motorcycles prefer to cross street facilities. The attributes of green facilities and shops on the path to school indicated that children who traveled by electric bicycle or motorcycle to school preferred the presence of shops and greenery such as lawns, flower ponds, and street trees on the path to/from school.

During the survey, the researchers found that most of the rural school-age children interviewed were transported to/from school by their parents on electric bicycles. In the survey on the preference of the built environment variables for children's school passages by electric bicycles, the highest significance was found for the range attribute of 1 to 2.5 km distance, and they also disliked the road environment with too high speed and too many intersections.

### Analysis of rural children's preference of private car in school travel environment

The effect of the distance attribute at the first and second levels is significant at 1% at conventional level and both are negative, given the choice of private car to and from school, indicating that when children choose private car to go to school, they do not prefer to travel by private car at distances < 500 m and distances between 500 m and 1 km, which may be due to children's consideration of factors such as time, cost, and convenience. The effect of distance attribute at the third level is positively significant at conventional level, when the distance to/from school is >1 km, they are willing to go to and from school willing to choose private car as the transportation. The effect of the first of the number of intersections attribute is significant at the conventional level of 1% and negative, indicating that children who travel to/from school by private car do not prefer road environments with more than five intersections and that too many intersections may prolong school travel time; the effects at the other two levels are not significant at the conventional level.

There is no significant difference in the attributes of sidewalks or bicycle lanes, speed, isolation facilities between motor vehicles and non-motor vehicles, and shops, which indicates that these attributes of the built environment for school travel have no impact on children taking private cars. The traffic light and crosswalk attributes have a 1% level of significance at the first level of impact with a high significance coefficient, which indicates that children who travel to/from school in private vehicles prefer to cross street facilities. The attributes of green facilities on the path to school indicate that children who travel by electric bicycle or motorcycle to school prefer to have greenery such as grass, flower ponds, and street trees on the path to/from school.

For children who go to/from school by private car, they prefer a built environment with fewer intersections, traffic lights with crosswalks and greenery, in addition to distance.

### Analysis synthesis

The four multinomial logit models analyzing the preferences of the school travel built environment for the four travel modes showed two constant terms. Only the constant term of the choice experimental model in the walking condition was negatively significant, while the constant terms of the remaining three choice experimental models were positively significant. This suggests that in addition to the attributes and levels set by this study, there are other attributes of the school travel built environment that can affect walking or bicycle, electric bicycle, private vehicle, etc.

## Conclusions and policy recommendations

The purpose of this study is to provide more insights on the relationship between the micro built environment attributes (like travel path attributes) and children's travel mode preference for school. To this end, a statement choice experiment was designed in rural areas of Chengdu, China. Supposing that rural school-age children want to walk, ride a bicycle, or take a private car to and from school, we invite them to point out which one they will choose under different street profiles. Compared with the judgment of specific road sections, the advantage of using the statement choice experiment is that we can control the covariance of attribute levels. Thus, all else being equal, the results reflect a more basic measure of children's preferences.

The research results show that, in addition to the general road related variables (distance, number of intersections passed, sidewalk/bicycle lane, traffic speed, isolation facilities between motorway and non-motorway, traffic lights and zebra crossings and other street crossing facilities), the greening and shop also have a significant impact on children who go to and from school by walking, bicycle, electric bicycle or motorcycle, and private car. For road related variables, the results show that children who commute to school on foot, bicycle, electric bicycle or motorcycle prefer to commute to school sections with sidewalks or bicycle lanes, few intersections, low traffic speed, green plants and shops.

In this study, children's preferences and differences for the school section under the four transportation modes are compared. For the distance attribute, children who walk to school or go to school by bike have a positive preference for shorter road sections, but children who walk prefer the road sections within 500 m, while children who go to school by bike prefer the road sections within 500 m to 1 km. For children who go to school by electric bicycle or motorcycle and private car, they will choose the road sections with larger distances. Children prefer a safer school travel built environment, such as a school travel roadway with fewer intersections, separated facilities, sidewalks or bike lanes, and well-developed crossing facilities.

Some of the results are consistent with the literature. Children who were physically exposed to environmental passages were more sensitive to green plants. Children's nature also leads them to have a significant positive preference for the school path with shops. The literature also shows that shops (retail stores) are having an impact on children's school travel (75, 80). In this study shops were found to be a highly preferred attribute level for children who walked, biked, or commuted to school by electric bicycle/motorcycle. The presence of greenery and sidewalks/bike lanes also influence children's school travel styles, which have been studied in most past studies (81–85). In this study, greenery and sidewalks/bike lanes were studied at both the presence and absence levels to show the impact on children's school travel styles. The traffic lights as one of the crossing facilities have been studied in past studies (86–89). However, zebra crossing, an important crossing facility, have been little studied together with traffic lights. This study includes both crossing facilities together and provides two preferences for them. The purpose of this study is to explore a healthy built environment for rural school-age children and to reduce parents' commuting pressure to and from school by improving the built environment for school travel and promoting public health. However, the results of the study may not comprehensively reveal the implied pattern of the built environment impacts on road choice for rural students due to long-standing constrains for a single case study (90, 91), more cases are suggested to further explore the pattern to acquire more insights in future studies.

Some advice based on the empirical results are listed here: relieving traffic congestion and chaos caused by private cars and electric bicycle to the vicinity of the school; providing a built environment for children to go to/from school alone; promoting active school traveling for rural children; increasing social interaction opportunities for children, and promoting low-carbon travel for rural residents. This research can provide some inspiration and reference for future rural road planning and design practice.

## Data availability statement

The raw data supporting the conclusions of this article will be made available by the authors, without undue reservation.

## Ethics statement

Ethical review and approval was not required for the study on human participants in accordance with the local legislation and institutional requirements. Written informed consent to participate in this study was provided by the participants' legal guardian/next of kin.

## Author contributions

Conceptualization and formal analysis: LH and YW. Data curation: LH, XD, and ML. Funding acquisition and writing—review and editing: TW. Investigation: LH and XD. Methodology and project administration: YW. Supervision: YW and TW. Writing—original draft: LH, XD, and TW. All authors have read and agreed to the published version of the manuscript. All authors contributed to the article and approved the submitted version.
